# Rescue of Progeria in Trichothiodystrophy by Homozygous Lethal *Xpd* Alleles

**DOI:** 10.1371/journal.pbio.0040322

**Published:** 2006-10-03

**Authors:** Jaan-Olle Andressoo, Judith Jans, Jan de Wit, Frederic Coin, Deborah Hoogstraten, Marieke van de Ven, Wendy Toussaint, Jan Huijmans, H. Bing Thio, Wibeke J van Leeuwen, Jan de Boer, Jean-Marc Egly, Jan H. J Hoeijmakers, Gijsbertus T. J van der Horst, James R Mitchell

**Affiliations:** 1 Medical Genetics Center, Department of Cell Biology and Genetics, Center of Biomedical Genetics, Erasmus Medical Center, Rotterdam, Netherlands; 2 Institut de Genetique et de Biologie et Cellulaire, Strasbourg, France; 3 Department of Clinical Genetics, Erasmus Medical Center, Rotterdam, Netherlands; 4 Department of Dermatology, Erasmus Medical Center, Rotterdam, Netherlands; 5 Department of Experimental Radiology, Erasmus Medical Center, Rotterdam, Netherlands; Genome Institute of Singapore, Singapore

## Abstract

Although compound heterozygosity, or the presence of two different mutant alleles of the same gene, is common in human recessive disease, its potential to impact disease outcome has not been well documented. This is most likely because of the inherent difficulty in distinguishing specific biallelic effects from differences in environment or genetic background. We addressed the potential of different recessive alleles to contribute to the enigmatic pleiotropy associated with *XPD* recessive disorders in compound heterozygous mouse models. Alterations in this essential helicase, with functions in both DNA repair and basal transcription, result in diverse pathologies ranging from elevated UV sensitivity and cancer predisposition to accelerated segmental progeria. We report a variety of biallelic effects on organismal phenotype attributable to combinations of recessive *Xpd* alleles, including the following: (i) the ability of homozygous lethal *Xpd* alleles to ameliorate a variety of disease symptoms when their essential basal transcription function is supplied by a different disease-causing allele, (ii) differential developmental and tissue-specific functions of distinct *Xpd* allele products, and (iii) interallelic complementation, a phenomenon rarely reported at clinically relevant loci in mammals. Our data suggest a re-evaluation of the contribution of “null” alleles to *XPD* disorders and highlight the potential of combinations of recessive alleles to affect both normal and pathological phenotypic plasticity in mammals.

## Abstract 2

Effects of mutations in *Xpd* were investigated in mice. Compound heterozygotes of otherwise homozygous lethal alleles demonstrated interallelic complementation and partial phenotypic rescue of XPD-related disease symptoms.

## Introduction

Interallelic complementation is defined as the ability of two differentially mutated alleles to function better together than either can on its own. Despite its near universality in lower organisms [1], its potential to contribute to clinical heterogeneity in human disease is seldom considered. Evidence of interallelic complementation at clinically relevant loci is limited to biochemical and cell-based studies of a handful of metabolic disorders with defects in enzymes including propinyl-CoA carboxylase [2], argininosuccinate lyase [3], galactose-1-phosphate uridylyltransferase [4], and methylmalonyl CoA mutase [5].

Compound heterozygotes are individuals carrying two different mutant alleles of the same gene. In the absence of a dominant (wild-type [wt]) allele, genetic interactions between recessive alleles (referred to here as “biallelic” effects) could result in different phenotypic outcomes including interallelic complementation. Although amelioration of disease symptoms by interallelic complementation would create an ascertainment bias in the clinic, the lack of evidence concerning interallelic complementation or other biallelic effects in human disease is likely caused by the difficulty in distinguishing such effects from environment and genetic background.

*XPD* encodes one of the two helicase components of basal transcription/DNA repair factor IIH (TFIIH), a ten-subunit, multifunctional complex that is essential for multiple processes, including basal transcription initiation and DNA damage repair via the nucleotide excision repair (NER) pathway [6,7]. Alterations in XPD resulting in defective TFIIH function are associated with UV-sensitive, multisystem disorders including xeroderma pigmentosum (XP), XP combined with Cockayne syndrome (CS), and trichothiodystrophy (TTD) [8–10]. XP is marked by sun-induced pigmentation anomalies and a greater than 1,000-fold elevation in skin cancer risk. Severe cases can also present with growth retardation and primary neurodegeneration [11]. CS and TTD, on the other hand, are segmental progeroid disorders characterised by progressive post-natal growth failure and primary demyelination resulting in severe neurodysfunction, but without a clear cancer predisposition [12–15]. Patients with TTD additionally display hallmark sulphur-deficient brittle hair and nails and scaling skin [13], resulting from a basal transcription defect in specific cell types [16,17]. A related disorder with the cancer predisposition of XP combined with the neurodevelopmental complications of CS (XPCS), although rare, has also been described [18].

Many *XPD* mutations are associated with an exclusive disease phenotype (e.g., XPD^R722W^ with TTD and XPD^R683W^ with XP) and are thus viewed as causative of the corresponding syndromes. Alleles not associated exclusively with one disorder are considered “likely null” alleles [19,20]. Some of these alleles fail to support viability in a haploid Schizosaccharomyces pombe yeast strain with a null mutation in the *XPD* homologue *rad15* and are thus considered devoid of significant biological activity [19]. This classification of alleles as either causative or null currently defines what we refer to as a “monoallelic” paradigm of *XPD* disease. However, the identification in recent years of XP complementation group D patients with atypical disease presentation, including symptoms of both XP and TTD [8], casts doubt on the ability of such a monoallelic paradigm to explain clinical heterogeneity in compound heterozygotes.

Previously, we generated a TTD mouse model (XPD^R722W^) that phenocopies the human syndrome [15,21]. Here we report the generation of additional mutant *Xpd* alleles that fail to support viability on their own but nevertheless ameliorate TTD-associated premature segmental ageing, cutaneous features, cellular DNA repair capacity, and UV survival when present in a compound heterozygote state.

## Results

### Generation of *Xpd* Compound Heterozygotes

We generated an *Xpd* knock-in allele with a point mutation encoding a single amino acid change (XPD^G602D^) found in the XPCS patient XPCS2 (Figure 1A–1C). mRNA expression from the targeted allele could be detected in embryonic stem cells by RT-PCR (Figure 1D), although expression was reduced approximately 5-fold relative to wt mRNA transcript levels as determined by Northern blotting of RNA from the testis of heterozygous animals (Figure 1E). Because patient XPCS2 was a hemizygote with mutant XPD protein (XPD^G602D^) expressed from a single allele, the corresponding mutation was expected to be viable in the homozygous state. However, homozygous mutant mice were not observed, neither amongst live births nor embryonic day 13.5 (E13.5) or E3.5 embryos (Table 1). The corresponding hypomorphic, mutant allele was thus designated as homozygous lethal (†XPCS). Homozygous lethality of the XPCS allele is likely due to reduced levels of expression of this essential protein as a result of gene targeting (Figure 1A) rather than to the mutation itself. *Xpd* ablation *(Xpd^KO^* ^/*KO*^ *)* is similarly incompatible with life beyond the earliest stages of embryogenesis [22]. Consistent with this interpretation, a different targeted *Xpd* mutation encoding XPD^R683W^, which is associated with XP in the homozygous state in humans, was similarly underexpressed and lethal in the homozygous state (designated as †XP allele) (Figure 1A–1C; Table 1; unpublished data). Also, a different targeting approach leading to the use of the native 3′UTR and removal of the *neo* gene resulted in normalisation of *Xpd^XPCS^* mRNA levels and viable homozygous *Xpd^XPCS/XPCS^* (XPD^G602D/G602D^) animals [23].

**Figure 1:**
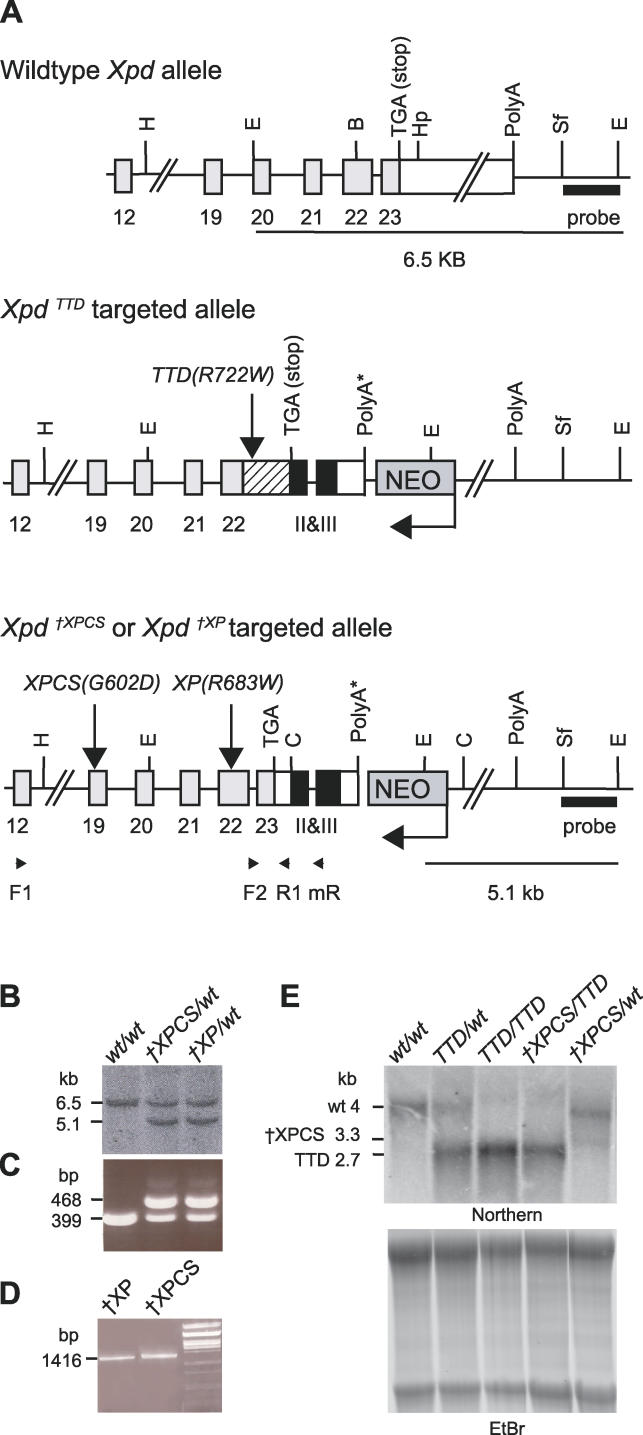
Targeting of the Mouse *Xpd* Gene (A) Schematic representation of the genomic structure and partial restriction map of the wt and targeted mouse *Xpd* loci. For the wt *Xpd* allele, shaded boxes represent coding regions of exons 12 and 19–23; the 3′UTR is represented by an open box. TGA indicates the translational stop codon; PolyA indicates the polyadenylation signal. For the *Xpd^TTD^* targeted allele, the 194–base pair (bp) human *XPD* cDNA fragment fused to exon 22 is indicated as a striped box including the TTD (R722W) mutation indicated by a vertical arrow. Chicken β-globin exons 2 and 3 including the 3′UTR are indicated as black boxes with corresponding Roman numerals followed by the β-globin polyadenylation signal (PolyA*). For the *Xpd^†XP^* and *Xpd^†XPCS^* targeted alleles, vertical arrows indicate XPCS (G602D-encoding) and XP (R683W-encoding) mutations in exons 19 and 22, respectively. The unique 3′ probe located outside the targeting construct is marked by a thick black line. Restriction sites: B, BamHI; C, ClaI; E, EcoRI; H, HindIII; Hp, HpaI; Sf, SfiI. (B) Southern blot analysis of EcoRI-digested genomic DNA from wt, *Xpd^†XPCS/wt^,* and *Xpd^†XP/wt^* recombinant embryonic stem cell clones hybridised with the 3′ probe depicted in (A). The wt allele yields a 6.5-kilobase (kb) fragment, whereas both targeted *Xpd^†XP^* and *Xpd^†XPCS^* alleles yield a 5.1-kb fragment. (C) Genotyping of wt and targeted alleles by PCR using primers F2, R1, and mR as indicated in (A) yields fragments of 399 bp and 468 bp, respectively. (D) RT-PCR detection of mRNA expression originating from the targeted *†XP* and *†XPCS* alleles in embryonic stem cell clones using primers F1 (hybridising outside the targeting construct) and mR as indicated in (A) results in a 1,416-bp fragment. (E) Northern blot analysis of total RNA isolated from testis of homozygous wt and *Xpd^TTD/TTD^,* heterozygous *Xpd^†XPCS/wt^* and *Xpd^TTD/wt^,* and compound heterozygous *Xpd^†XPCS/TTD^* mice as indicated. Hybridisation with a 1.4-kb mouse *Xpd* cDNA probe detects mRNAs of 4, 3.3, and 2.7 kb from wt, *Xpd^†XPCS^,* and *Xpd^TTD^* alleles, respectively. An ethidium bromide (EtBr)–stained gel showing the amount of total RNA loaded is shown below.

**Table 1:**
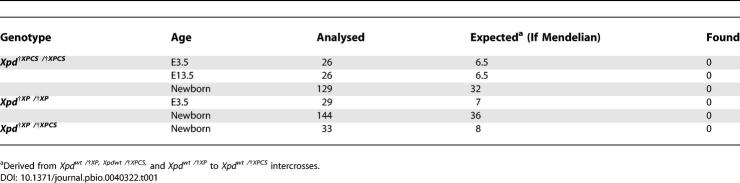
Frequency of *Xpd^†XP/†XP,^* *Xpd^†XPCS/†XPCS^,* and Compound Heterozygous *Xpd^†XP/†XPCS^* Embryos and Offspring

### “Null” Allele Can Alleviate Developmental Delay, Skin, and Hair Features of TTD

To test the potential of a homozygous lethal “null” allele to nevertheless contribute to organismal phenotype, we combined an *Xpd^†XPCS^* allele with a viable *Xpd^TTD^* allele by crossing the corresponding heterozygous animals. Similar to hemizygous TTD mice carrying one true *Xpd* knockout allele *(Xpd^TTD/KO^),* compound heterozygous *Xpd^TTD/†XPCS^* mice were born at the expected Mendelian frequencies. Expression from the *Xpd^†XPCS^* allele was also reduced in the testis of compound heterozygous animals, whereas expression from the *Xpd^TTD^* allele was increased relative to wt by ~5-fold (Figure 1E). Because of a lack of available antibodies and the inability to distinguish amongst various mutant forms of XPD differing only by single amino acid substitutions, we were unable to ascertain the relative amount of XPD protein from the different alleles.

Despite reduced levels of mRNA expression, the homozygous lethal *Xpd^†XPCS^* allele ameliorated multiple *Xpd^TTD^*-associated disease symptoms in compound heterozygous *Xpd^TTD/†XPCS^* animals including the hallmark brittle hair and cutaneous features fully penetrant in homo- and hemizygous TTD mice (Figure 2A–2C). In marked contrast to *Xpd^TTD/TTD^* (and *Xpd^TTD/KO^*) mice, which display complete hair loss in the first hair cycle and partial hair loss in subsequent cycles throughout their lives [21], compound heterozygous *Xpd^TTD/†XPCS^* mice displayed some hair loss only during the first hair cycle and only locally at the back (Figure 2A). Scanning electron microscope analysis of *Xpd^TTD/†XPCS^* hair revealed an almost normal appearance, with TTD-like features such as broken hairs found only at very low frequency (unpublished data). Amino acid analysis confirmed that cysteine levels in the hair of the *Xpd^TTD/†XPCS^* mice were significantly higher than in *Xpd^TTD/TTD^* animals, but remained below the wt level (Figure 2C). TTD hemizygotes *(Xpd^TTD/KO^)* do not display significant differences in cutaneous features and longevity relative to homozygous *Xpd^TTD/TTD^* mice [21].

**Figure 2:**
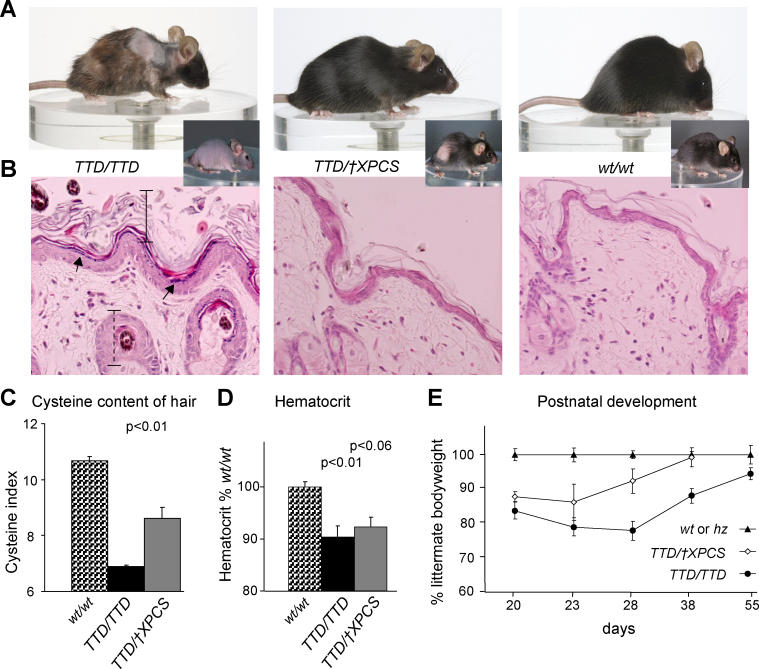
Partial Rescue of TTD Cutaneous, Blood, and Developmental Phenotypes in Compound Heterozygous *Xpd^TTD/†XPCS^* Mice (A) Photographs of 5-mo-old homozygous *Xpd^TTD/TTD^,* compound heterozygous *Xpd^TTD/†XPCS^,* and wt mice. Insets: images of first-round hair loss. (B) Histological analysis of the skin of *Xpd^TTD/TTD^, Xpd^TTD/†XPCS^,* and wt mice. TTD-associated acanthosis (thicker epidermis, indicated by solid vertical line), pronounced granular layer (indicated by arrows), and sebacious gland hyperplasia (indicated by dotted vertical line) were absent in the epidermis of *Xpd^TTD/†XPCS^* and wt mice. Magnification 400×. (C) Cysteine content of hair from wt, *Xpd^TTD/TTD^,* and *Xpd^TTD/†XPCS^* mice. The *p*-value indicates significant differences between mutants and wt, as well as between *Xpd^TTD/TTD^* and *Xpd^TTD/†XPCS^* mice. Error bars indicate standard error of the mean (SEM). (D) Hematocrit values from blood of *Xpd^TTD/TTD^* and *Xpd^TTD/†XPCS^* mice. The *p*-values indicate the significance of the difference relative to wt. Error bars indicate SEM. (E) Body weights of developing *Xpd^TTD/TTD^* and *Xpd^TTD/†XPCS^* mice after weaning plotted as a percentage of the weight of age-matched control wt and heterozygote (hz) littermates (set at 100%). Error bars indicate SEM.

Other prominent TTD features in the epidermis, including acanthosis (thickening of the layer of the nucleated cells), hyperkeratosis (prominent thickening of the cornified layer), and pronounced granular layer and sebacious gland hyperplasia (causing greasy appearance of the hair), were absent in the skin of *Xpd^TTD/†XPCS^* mice, as established by blind microscopic examination of skin sections (Figure 2B). Furthermore, anaemia and developmental delay present in patients with TTD [24] and in *Xpd^TTD/TTD^* mice [15] were both partially rescued in compound heterozygous *Xpd^TTD/†XPCS^* mice (Figure 2D and 2E).

### Rescue of Progeroid Features in TTD Mice by Homozygous Lethal *Xpd* Alleles

Because patients with TTD, XPCS, and CS (but not XP) and the corresponding mouse models share similar accelerated progeroid symptoms [12,13,15,23], we next addressed ageing-related parameters in compound heterozygous mice (Figure 3). Whereas *Xpd^TTD/TTD^* animals show reduced bone mineral density as an indication of the early onset of osteoporosis before ~14 mo of age [15], tail vertebrae from compound heterozygous *Xpd^TTD/†XPCS^* mice were comparable to wt even at 20 mo of age (Figure 3B and 3C). Furthermore, whereas *Xpd^TTD/TTD^* mice developed kyphosis earlier than wt animals (onset ~3 mo versus 12–20 mo), compound heterozygous *Xpd^TTD/†XPCS^* mice did not (Figure 3B). Overall appearance and body weight curves revealed that TTD-associated age-related premature cachexia and lack of general fitness were fully rescued in compound heterozygous *Xpd^TTD/†XPCS^* mice (Figure 3A and 3D). Finally, the life span of compound heterozygotes was extended relative to *Xpd^TTD/TTD^* mice (Table 2).

**Figure 3:**
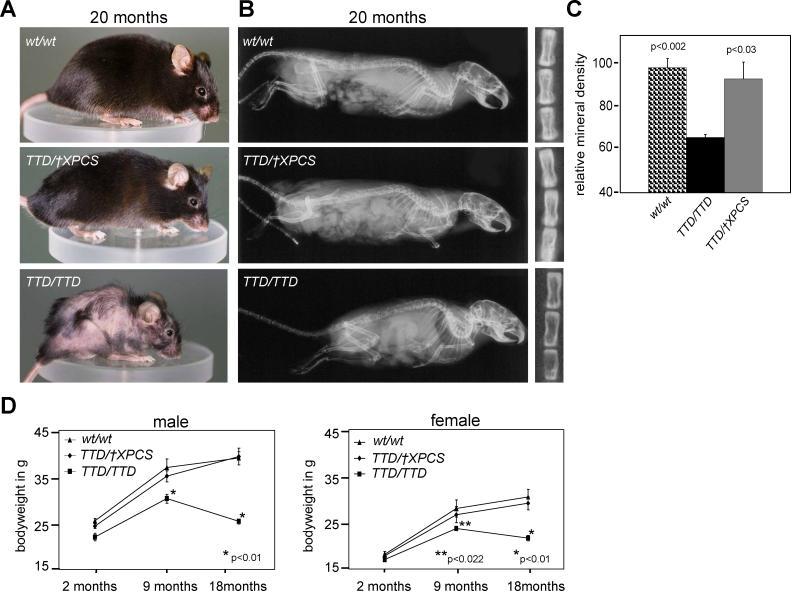
Rescue of TTD-Associated Segmental Progeroid Features in Compound Heterozygous *Xpd^ TTD/†XPCS^* Mice (A) Photographs of 20-mo-old wt, compound heterozygous *Xpd^TTD/†XPCS^,* and homozygous *Xpd^TTD/TTD^* mice. Note the extreme cachexia (lack of subcutaneous fat) in the *Xpd^TTD/TTD^* mouse and the absence of this phenotype in wt and *Xpd^TTD/†XPCS^* mice. (B) Radiographs of 20-mo-old male wt, *Xpd^TTD/†XPCS^,* and *Xpd^TTD/TTD^* mice. Ageing *Xpd^TTD/TTD^* mice develop kyphosis (curvature of the spinal column) and reduction of bone mineral density as shown in the 6–8 segment of the tail vertebrae counted from the pelvis (see close-up at right). Note the absence of these features in the *Xpd^TTD / † XPCS^* mouse. (C) Quantification of relative bone mineral density of tail vertebrae from 20-mo-old male wt (*n* = 3), *Xpd^TTD/†XPCS^* (*n* = 4), and *Xpd^TTD/TTD^* (*n* = 3) mice. The *p*-values indicate the significance of the difference relative to *Xpd^TTD/TTD^*. Error bars indicate SEM. (D) Body weight curves as a function of time. Note that the age-dependent cachexia observed in *Xpd^TTD/TTD^* mice was rescued in both male and female *Xpd^TTD / †XPCS^* mice. Significant differences between wt and *Xpd^TTD/TTD^* but not between wt and *Xpd^TTD/†XPCS^* mice were observed at 9 and 18 mo of age as indicated by asterisks. Error bars indicate SEM.

**Table 2:**
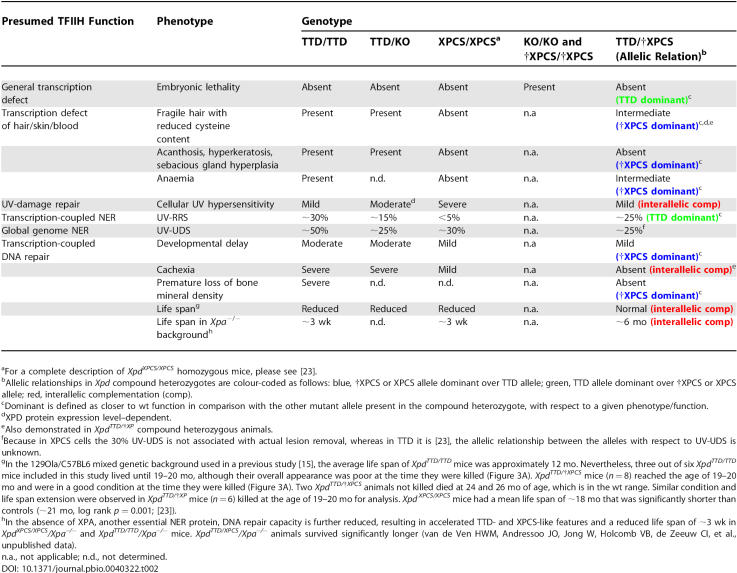
Pleiotropic *Xpd* Biallelic Effects in Mice and Cells

To determine whether the homozygous lethal *Xpd^†XPCS^* allele was unique in its ability to ameliorate symptoms associated with the *Xpd^TTD^* allele, we generated compound heterozygous *Xpd^TTD/†XP^* mice by crossing the corresponding heterozygous animals. Similar to the *Xpd^ †XPCS^* allele, the homozygous lethal *Xpd^ †XP^* allele rescued cutaneous symptoms including hair loss (except locally during the first round; unpublished data), reduced cysteine content (cysteine index 9.3 ± 0.9 standard deviation [87% of wt], *p* = 0.01 versus TTD), ageing-associated premature cachexia (males and females were 36.1 ± 6.4 g [93% of wt] and 39.2 ± 3.2 g [116% of wt], respectively), and reduced life span (Table 2). Taken together, these data indicate that two independent alleles, which on their own are unable to support viability (Table 1), were nonetheless able to ameliorate TTD-associated phenotypes in vivo (Table 2).

### Molecular Mechanisms of Biallelic Effects

We next turned to UV-based cellular assays including unscheduled DNA synthesis after UV irradiation (UV-UDS), recovery of RNA synthesis after UV irradiation (UV-RRS), and UV survival, which report on the NER subpathways (global genome NER and transcription-coupled NER) and total NER, respectively. In none of these assays was the response to UV improved in compound heterozygotes relative to TTD homozygotes (Figure 4A–4C). However, unlike the in vivo TTD phenotypes described above, in which *Xpd^TTD/TTD^* and *Xpd^TTD/KO^* animals were indistinguishable, *Xpd^TTD^* dosage effects were observed in UV survival, UV-UDS, and UV-RRS, indicating that cellular parameters as measured in fibroblasts here do not always correlate with the phenotype at the level of the intact organism. *Xpd^TTD/KO^* hemizygous cells were thus used as the baseline on which to compare the activity of compound heterozygous cells. Relative to *Xpd^TTD/KO^* hemizygote cells, UV survival was improved by the homozygous lethal *Xpd^†XPCS^* allele in *Xpd^TTD/†XPCS^* compound heterozygous cells and to a lesser degree by the *Xpd^†XP^* allele (Figure 4A). Because of embryonic and cellular lethality, we were unable to test UV survival associated exclusively with the *Xpd^†XPCS^* or *Xpd^†XP^* alleles. However, homozygous *XPD^XP^* (XPD^R683W^) and hemizygous *XPD^XPCS^* (XPD^G602D^) human cells are known to be highly sensitive to UV [19,25], as are cells from a homozygous viable *Xpd^XPCS/XPCS^* (XPD^G602D/G602D^) mouse model (Figure 4A, dotted line) [23]. Thus, the survival of *Xpd^TTD/†XPCS^* (and *Xpd^TTD/†XP^*) cells likely represents a level of UV resistance that neither mutant allele can impart on its own (Table 2). Significant effects of compound heterozygosity on NER subpathways relative to *Xpd^TTD/KO^* cells were observed in *Xpd^TTD/†XP^* cells but only for UV-UDS activity. Finally, none of the mutant TFIIH combinations (carrying alterations associated with TTD [XPD^R722W^], XPCS [XPD^G602D^], or XP [XPD^R683W^]) exhibited synergism in an in vitro NER reaction reconstituted with different mutant TFIIH complexes (Figure 4D). Taken together, these data are consistent with interallelic complementation of UV sensitivity in cells but underscore the lack of any correlation between UV-related repair characteristics and TTD progeroid phenotypes in animal models.

**Figure 4:**
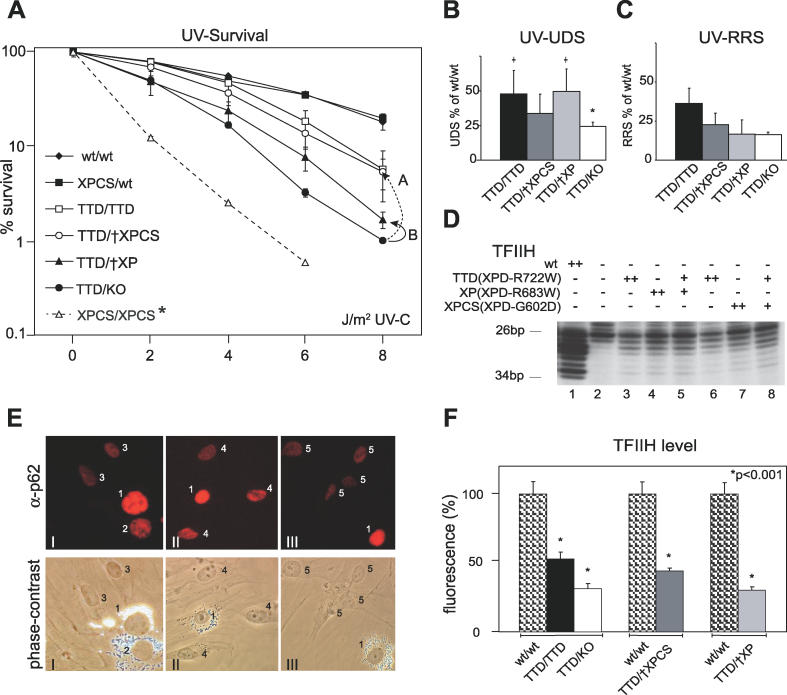
TFIIH Functions and Mechanisms of XPD-Associated Disease Pleiotropy (A) Cellular survival after UV irradiation. Rescue of hemizygous *Xpd^TTD/KO^* survival by *Xpd^†XPCS^* and *Xpd^†XP^* alleles is illustrated by arrows marked A and B, respectively. UV survival of homozygous *Xpd^XPCS/XPCS^* cells (asterisk) from the normally expressed viable allele *(Xpd^XPCS^)* is depicted by a dotted line. Survival curves represent an average of four independent experiments; 1–2 cell lines per genotype were included in each experiment. Error bars indicate SEM between experiments. (B) UV-UDS, a measure of global genome repair. Number of experiments: *n* = 15 *(Xpd^TTD/TTD^), n* = 6 *(Xpd^TTD/KO^), n* = 4 *(Xpd^TTD/†XPCS^* *)*, *n* = 2 *(Xpd^TTD/†XP^* *);* 1–2 cell lines per genotype were included in each experiment. The asterisk indicates significant difference with *Xpd^TTD/TTD^;* crosses indicate significant differences with *Xpd^TTD/KO^*. (C) UV-RRS, a measure of transcription-coupled repair of UV-induced lesions. Number of experiments: *n* = 7 *(Xpd^TTD/TTD^), n* = 2 *(Xpd^TTD/KO^), n* = 4 *(Xpd^TTD/†XPCS^* *)*, n = 2 *(Xpd^TTD/†XP^* *)*; 1–2 cell lines per genotype were included in each experiment. (D) Incision/excision activity of combinations of altered TFIIH complexes in a reconstituted NER reaction. Equal amounts of single or mixed populations of recombinant TFIIHs (containing XPD, XPB, p62, p52, His-p44, Flag-p34, cdk7, cyclin H, Mat1, and p8) were mixed with recombinant XPG, XPF/ERCC1, XPC/hHR23B, RPA, and a radiolabelled synthetic NER substrate. The excision products (26–34 nucleotides in length) were visualised at nucleotide resolution on a denaturing polyacrylamide gel as indicated . Note the weak activity corresponding to each single and combined TFIIH complex (lanes 3–8) relative to the wt (lane 1) and negative controls (lane 2). (E) *Xpd* dose-dependent reduction of TFIIH in homozygous *Xpd^TTD/TTD^,* hemizygous *Xpd^TTD/KO^,* and compound heterozygous *Xpd^TTD/†XPCS^* and *Xpd^TTD/†XP^* cells by comparative immunofluorescence of the p62 subunit of TFIIH. Roman numerals represent different microscopic slides and Arabic numerals different cell lines labelled as follows: (I) wt cells (1) labelled with 2-μm beads, *Xpd^TTD/TTD^* cells (2) with 0.79-μm beads, and *Xpd^TTD/KO^* cells (3) with no beads; (II) wt cells (1) labelled with 0.79-μm beads and *Xpd^TTD/†XPCS^* cells (4) with no beads; and (III) wt cells (1) labelled with 0.79-μm beads and *Xpd^TTD/†XP^* cells (5) with no beads. (F) Quantification of immunofluorescent signal from at least 50 nuclei per cell line and 2–6 experiments per genotype. Bars representing cells analysed on the same microscopic slide are depicted side by side, with wt set at 100%. The *p*-value indicates minimum significant difference between wt and the indicated cell lines analysed on the same microscopic slide within one experiment.

Next we asked whether the *Xpd^†XPCS^* and *Xpd^†XP^* alleles, despite decreased mRNA levels, ameliorated TTD symptoms by increasing overall TFIIH levels in compound heterozygous *Xpd^TTD/ †XPCS^* and *Xpd^TTD/ †XP^* cells. Previously, using comparative immunohistochemistry, we and others have shown an up to 70% reduction of TFIIH levels in primary fibroblasts from patients with TTD compared with wt controls due to reduced stability [16,17]. Despite overexpression of mRNA from the *Xpd^TTD^* allele relative to the wt allele (Figure 1E), TFIIH protein levels were reduced by 50% in primary mouse *Xpd^TTD/TTD^* fibroblasts (Figure 4E and 4F), thereby mimicking the situation in human patients with TTD. In accordance with the gene dosage, a further reduction of up to 70% of the wt level was observed in hemizygous *Xpd^TTD/KO^* cells. Consistent with low mRNA expression levels, neither the *Xpd^†XPCS^* nor the *Xpd^†XP^* allele was able to restore TFIIH abundance to wt levels in *Xpd^TTD^* compound heterozygote cells (Figure 4E and 4F). Thus, the improved UV survival observed in compound heterozygote cells (Figure 4A) and likely the rescue of TTD progeroid symptoms (Figure 3) were not due to normalisation of TFIIH levels, suggesting a qualitative rather than a quantitative effect on these phenotypes in vivo.

In contrast, the level of XPCS mRNA expression did affect the ability of the encoded protein (XPD^G602D^) to restore the TTD hair phenotype to normal. Notably, *Xpd^TTD/ †XPCS^* animals had a partial TTD hair phenotype, correlating with low levels of *Xpd^†XPCS^* expression, whereas *Xpd^TTD/XPCS^* animals had wt hair, correlating with normal expression levels from the viable *Xpd^XPCS^* allele (Table 2 and unpublished data). Thus, the range of expression levels from these two mutant alleles affected their ability to complement some phenotypes (hair). An overview of the functional relationships between *Xpd* alleles, phenotypes, and the presumed underlying TFIIH function in mice and cells is presented in Table 2.

## Discussion

### Dissection of Biallelic Effects from other Determinants of Phenotype

Although phenotypic consequences, referred to here as biallelic effects, resulting from two different mutant alleles in compound heterozygote patients have been postulated, such effects have historically been difficult to distinguish from the influence of environment and genetic background. We used a genetically defined mammalian model system under controlled environmental conditions to reveal phenotypic effects attributable specifically to combinations of differentially mutated *Xpd* alleles.

The observed biallelic effects were of three general types. In the first, the allele associated in a homozygous state with a phenotype closer to wt singularly determined the phenotypic outcome, a phenomenon widely known in human recessive disease. Because these *Xpd* alleles functioned at or near wt levels with respect to a particular function, we call these effects “dominant”. Such alleles can also be referred to as “separation of function” alleles, because they allow dissection of the roles of multifunctional proteins in specific phenotypes.

Secondly, highlighting the potential relevance of current findings to all diploid organisms including humans was the observation that in one compound heterozygous animal, the *Xpd* allelic relationship could shift from *A*_dominant_ *|a*_recessive_ to *A*_recessive_ *|a*_dominant_ with respect to different phenotypes in a time-dependent and tissue-specific manner (see below and Table 2).

In the third type of biallelic effect, known as interallelic complementation, two mutant alleles produced a phenotype closer to wt than either could alone in a homo- or hemizygous state. As summarised in Table 2, examples of all types of biallelic effects were observed in a variety of *Xpd*-associated phenotypes, ranging from brittle hair to segmental progeria.

### TFIIH in Transcription and Repair: Mechanisms of XPD Disease Pleiotropy

We observed differences in the ability of *Xpd^TTD^* versus homozygous lethal *Xpd^†XPCS^* and *Xpd^†XP^* alleles to function in two transcription-related phenotypes separated in the organism by both time and space: embryonic lethality and terminal differentiation of enucleating skin and blood cells. The preblastocyst-stage homozygous lethality shared by the *Xpd^KO^, Xpd^†XPCS^,* and *Xpd^†XP^* alleles most likely reflects a defect in basal transcription that is incompatible with life. In *Xpd^TTD/ †XPCS^* and *Xpd^TTD/ †XP^* compound heterozygous mice, embryonic lethality was fully rescued by the *Xpd^TTD^* allele. Because embryonic lethality was also fully rescued in *Xpd^TTD/KO^* hemizygous mice, the *Xpd^TTD^* allele can be considered as wt and thus dominant to each of the homozygous lethal alleles *(Xpd^KO^, Xpd^†XPCS^,* and *Xpd^†XP^)* with respect to this particular phenotype (Table 2).

TTD-specific cutaneous and anaemic features, on the other hand, are thought to result from a specific kind of transcriptional insufficiency caused by depletion of unstable TFIIH during the terminal differentiation of skin, hair-shaft, and blood cells [16,24]. In compound heterozygous mice, both homozygous lethal *Xpd^†XPCS^* and *Xpd^†XP^* alleles were able to alleviate *Xpd^TTD^*-specific cutaneous and anaemic features and can thus be defined as dominant over the *Xpd^TTD^* allele with respect to these phenotypes. We conclude that the defects leading to embryonic lethality and aberrant terminal differentiation of the skin, hair, and blood represent two qualitatively and/or quantitatively different transcriptional deficiencies. During early embryonic development, *Xpd^TTD^* is dominant over the *Xpd^†XPCS^* and *Xpd^†XP^* alleles, whereas later in the ontogenesis of skin, hair-shaft, and blood cells, the situation is reversed.

In its role in the repair of UV photolesions, the *Xpd^†XPCS^* allele imparted a clear UV survival benefit over a single *Xpd^TTD^* allele or two *Xpd^XPCS^* alleles independent of expression levels, which is consistent with interallelic complementation. However, the observation that no other cellular or biochemical UV-related parameters were improved in *Xpd^TTD/ †XPCS^* argues against complementation of this repair activity in the rescue of TTD progeroid symptoms in vivo.

### Interallelic Complementation and XPD Function

What does interallelic complementation tell us about the mechanism of XPD function? Interallelic complementation is most often observed in multimeric proteins with multiple functional domains. Unfortunately, the structure–function relationship between disease-causing mutations and XPD functional domains, including detailed structural information on XPD or even its stoichiometry within TFIIH, remains unknown. However, based on the ability of cell extracts that are defective in two different TFIIH components (XPD and XPB) to complement NER activity in vitro [26], it is likely that TFIIH (or its components) can either multimerise or exchange at least during the NER reaction. Furthermore, XPD is known to be a “loosely bound” subunit of TFIIH [27]. We thus envisage the molecular mechanism of interallelic complementation to involve the exchange of XPD molecules within the TFIIH complex or turnover of TFIIH complexes containing different XPD molecules at the site of DNA damage during the course of the global genome as well as transcription-coupled repair of either UV-induced or endogenous DNA damage.

### A Biallelic Paradigm for XPD Disorders

Recently, proteins originating from presumed null alleles were biochemically characterised as inactive in basal transcription [27], providing an explanation as to why these alleles failed to rescue lethality in haploid S. pombe with a null mutation in the *XPD* homologue *rad15* [19]. Our data suggest that certain presumed null alleles, although unable on their own to support basal transcription, may in fact have a substantial impact on disease outcome in compound heterozygous humans, as they do in mouse models.

Clinical evidence in support of this hypothesis comes from a number of XP complementation group D patients that do not fit within the framework of the current monoallelic paradigm of XPD disorders (Figure 5). In contrast to two hemizygous *XPD^XPCS^* patients carrying the XPD^G47R^- or XPD^R666W^-encoding alleles who died of the disease before 2 y of age, two compound heterozygous *XPD^XPCS^* patients carrying the same XPD^G47R^- or XPD^R666W^-encoding alleles in addition to the presumed null XPD^L461V+del716−730^ both had considerably milder disease symptoms and survived more than ten times longer (A. Lehmann, personal communication) (Figure 5). Compound heterozygosity is also associated with the recently reported combination XP and TTD (XPTTD) syndrome [8]. Similar to the *Xpd^TTD/†XPCS^* and *Xpd^TTD/†XP^* mice described here, both patients with XPTTD described so far had intermediate hair cysteine values. Furthermore, XPTTD patient XP38BR carried a “causative” TTD mutation in one allele and a novel point mutation encoding XPD^L485P^ in the other. Although the XPD^L485P^-encoding allele fails to complement viability in the haploid *S. pombe rad15* deletion strain and is thus interpretable as a null allele [8], we nonetheless suggest that the combined XPTTD phenotype in this patient involves phenotypic contributions from both alleles. Taken together, these data suggest a shift to a biallelic paradigm for compound heterozygous patients in XP complementation group D.

**Figure 5:**
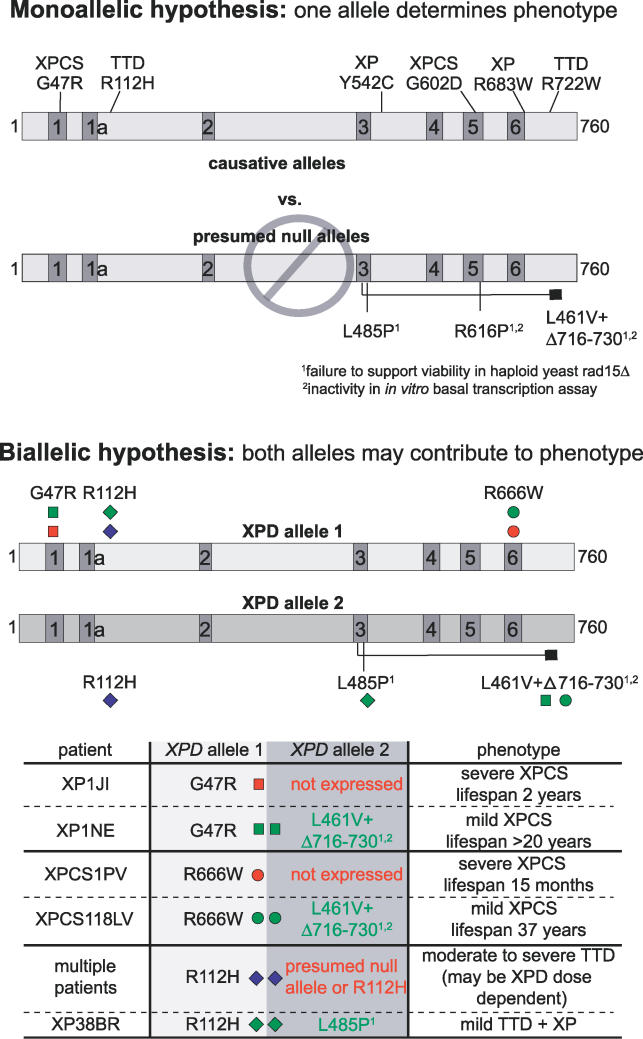
Genotype–Phenotype Relationships in XPD Disorders According to the current monoallelic hypothesis, phenotype is determined solely by the causative allele product. If a second, different allele is present, it is considered a functional null. There is a lack of any correlation between the site of the *XPD* mutation and the resulting disorder. We propose a biallelic hypothesis for compound heterozygotes in which both alleles can contribute to the phenotype. Examples of compound heterozygous patients in which a second, presumed null allele is likely to contribute to disease outcome are provided above in comparison to corresponding homo- or hemizygous patients with the same causative allele. Numbers in the schematic of the protein indicate the helicase domains.

### Potential of Combined Recessive Alleles to Affect Phenotypic Diversity in Mammals

In humans, the clinical relevance of biallelic effects such as interallelic complementation remains unknown. Although interallelic complementation between two endogenous mutant alleles has been described in cells from a compound heterozygous patient with methylmalonic acidaemia, no observable effects on disease outcome were noted in the patient [28]. Thus, to the best of our knowledge, the amelioration of progeroid features observed here is the first in vivo demonstration in compound heterozygous animals of interallelic complementation relevant to a human disease. Keeping in mind that the ~1,200 alleles known to exist for the *CTRF* gene implicated in the common autosomal recessive disorder cystic fibrosis alone [29] can theoretically result in ~700,000 different allelic combinations, the potential number of allelic combinations of different recessive mutations and single nucleotide polymorphisms genome-wide is currently incalculable. We suggest biallelic effects as a previously underestimated yet important variable in considering genotype–phenotype relationships from autosomal recessive disease to normal phenotypic diversity in mammals. Extension of the above concept implies that recessive mutations can enter evolutionary selection in F1 provided that the second allele carries a different recessive alteration. Finally, our data highlight the potential of clinically relevant alleles previously designated as null, with little or no detectable expression or activity, to nonetheless contribute to phenotype.

## Materials and Methods

### Derivation and analysis of mutant mice.

Generation of *Xpd^TTD^* (XPD^R722W^) and *Xpd^TTD/KO^* mice has been described previously [21,22]. A detailed description of the generation of targeting constructs for *Xpd^†XPCS^* and *Xpd^ †XP^* alleles carrying mutations encoding the G602D and R683W alterations will be provided upon request. Chimeric mice and mouse embryonic fibroblasts were generated according to standard procedures. Haematoxylin and eosin staining was performed according to standard procedures. Amino acid analysis was conducted as described in [21]. Blood values were analysed using Animal Blood Counter Vet (ABX Diagnostix, Montpellier, France). Radiographs were taken, and relative bone mineral density was calculated as described in [15]. Mice used in this study were in a 129Ola/C57BL6 mixed background unless noted differently. All experiments involving mice were judged and approved by the national committee for genetic identification of organisms and the animal ethical committee, and were conducted according to national and international guidelines.

### UV sensitivity, UV-UDS, UV-RRS, and TFIIH incision/excision activity.

UV survival, UV-UDS, and UV-RRS assays were performed as described previously [21,30]. For UV-RRS, average values from the representative experiment containing two wt, three *Xpd^TTD/TTD^,* two *Xpd^TTD/XPCS^,* and one *Xpd^TTD/XP^* cell line are presented. The ~48% UV-UDS value presented in this study for *Xpd^TTD/TTD^* cells differs from our previously published data of 25% UV-UDS [21], possibly because of the high variability intrinsic to the assay or routine variations in the cell culture conditions. For the incision/excision activity assay, recombinant TFIIH was prepared and assayed as described previously [27].

### Comparative immunofluorescence.

Latex bead labelling and comparative immunofluorescence analysis of the p62 subunit of the TFIIH was performed as described previously [16,17] using primary mouse embryonic fibroblasts at passages 2–5. Two or more cell lines per genotype (except for the *Xpd^TTD/†XP^* cells, in which only one cell line was used in repeated experiments) were used, and experiments were repeated 2–6 times per genotype.

## Abbreviations

bp: base pair
CS: Cockayne syndrome
E[number]: embryonic day [number]
kb: kilobase
NER: nucleotide excision repair
SEM: standard error of the mean
TFIIH: basal transcription/DNA repair factor IIH
TTD: trichothiodystrophy
UV-RRS: recovery of RNA synthesis after ultraviolet irradiation
UV-UDS: unscheduled DNA synthesis after ultraviolet irradiation
wt: wild-type
XP: xeroderma pigmentosum
XPCS: xeroderma pigmentosum combined with Cockayne syndrome
XPTTD: combination xeroderma pigmentosum and trichothiodystrophy
